# Whole-body uptake classification and prostate cancer staging in ^68^Ga-PSMA-11 PET/CT using dual-tracer learning

**DOI:** 10.1007/s00259-021-05473-2

**Published:** 2021-07-07

**Authors:** Nicolò Capobianco, Ludovic Sibille, Maythinee Chantadisai, Andrei Gafita, Thomas Langbein, Guenther Platsch, Esteban Lucas Solari, Vijay Shah, Bruce Spottiswoode, Matthias Eiber, Wolfgang A. Weber, Nassir Navab, Stephan G. Nekolla

**Affiliations:** 1 Technische Universität München, Munich, Germany; 2 Siemens Healthcare GmbH, Erlangen, Germany; 3 Siemens Medical Solutions USA, Inc., Knoxville, TN USA; 4 School of Medicine, Department of Nuclear Medicine, Technische Universität München, Munich, Germany; 5 Faculty of Medicine, King Chulalongkorn Memorial Hospital, The Thai Red Cross Society, Chulalongkorn University, Bangkok, Thailand; 6 Computer Aided Medical Procedures (CAMP), Technische Universität München, Munich, Germany

**Keywords:** Prostate cancer, Staging, PSMA, PET/CT, Deep learning, miTNM

## Abstract

**Purpose:**

In PSMA-ligand PET/CT imaging, standardized evaluation frameworks and image-derived parameters are increasingly used to support prostate cancer staging. Clinical applicability remains challenging wherever manual measurements of numerous suspected lesions are required. Deep learning methods are promising for automated image analysis, typically requiring extensive expert-annotated image datasets to reach sufficient accuracy. We developed a deep learning method to support image-based staging, investigating the use of training information from two radiotracers.

**Methods:**

In 173 subjects imaged with ^68^Ga-PSMA-11 PET/CT, divided into development (121) and test (52) sets, we trained and evaluated a convolutional neural network to both classify sites of elevated tracer uptake as nonsuspicious or suspicious for cancer and assign them an anatomical location. We evaluated training strategies to leverage information from a larger dataset of ^18^F-FDG PET/CT images and expert annotations, including transfer learning and combined training encoding the tracer type as input to the network. We assessed the agreement between the N and M stage assigned based on the network annotations and expert annotations, according to the PROMISE miTNM framework.

**Results:**

In the development set, including ^18^F-FDG training data improved classification performance in four-fold cross validation. In the test set, compared to expert assessment, training with ^18^F-FDG data and the development set yielded 80.4% average precision [confidence interval (CI): 71.1–87.8] for identification of suspicious uptake sites, 77% (CI: 70.0–83.4) accuracy for anatomical location classification of suspicious findings, 81% agreement for identification of regional lymph node involvement, and 77% agreement for identification of metastatic stage.

**Conclusion:**

The evaluated algorithm showed good agreement with expert assessment for identification and anatomical location classification of suspicious uptake sites in whole-body ^68^Ga-PSMA-11 PET/CT. With restricted PSMA-ligand data available, the use of training examples from a different radiotracer improved performance. The investigated methods are promising for enabling efficient assessment of cancer stage and tumor burden.

**Supplementary Information:**

The online version contains supplementary material available at 10.1007/s00259-021-05473-2.

## Introduction

Accurate staging has a pivotal role in the management of prostate cancer, a disease with generally favorable outcome when confined to the prostate, while having poor prognosis if metastasized at the time of diagnosis [1]. As a plethora of management strategies is available, ranging from watchful waiting to localized and systemic treatments, reliable information on the disease spread pattern and overall burden is crucial in the clinical decision-making process [2]. While the gold standard for prostate cancer staging remains histopathology, imaging is increasingly being utilized as noninvasive assessment [3]. Notably, prostate-specific membrane antigen (PSMA)-targeted PET/CT has shown high accuracy, superior to other imaging modalities, for primary staging of high-risk prostate cancer patients [4, 5] as well as for staging after biochemical recurrence [6, 7]. The ^68^Ga-PSMA-11 compound manufactured by the University of California, San Francisco, and the University of California, Los Angeles, has recently received approval from the U.S. Food and Drug Administration.

In addition to procedure guidelines [8], pitfalls reviews [9–11], and case reports [12, 13], standardized reporting frameworks for PSMA-ligand PET have been proposed to support replicable and rigorous image assessment [14–16]. Moreover, the use of quantitative image-derived biomarkers, such as the total tumor volume, has shown promising results for risk stratification and response assessment [17–20]. Nevertheless, the application in clinical routine of detailed reporting schemes and image-derived biomarkers remains labor intensive, subject to error, and operator dependent in cases where a high number of manual measurements are required, such as when categorical or quantitative variables have to be determined for all suspected lesions. In this context, the use of semi-automated and automated image analysis methods is promising to support accurate, reproducible, and time-efficient assessment.

Recently, semi-automated and automated methods for image analysis in ^68^Ga-PSMA-11 PET/CT have been developed. A convolutional neural network (CNN) was trained to predict the PSMA-ligand PET positivity status of lymph nodes from CT alone [21], showing a performance comparable to trained radiologists. To support semi-automated quantification of tumor burden, masks of organs that exhibit physiological uptake and bone were obtained from CT images using thresholding methods [22, 23], machine learning methods [24], and deep learning methods [18]. While the CT information alone can be used to aid semi-automated identification and anatomical localization of suspicious elevated uptake sites, including the PET information in a machine learning system for whole-body PSMA-ligand image analysis could be beneficial. In particular, the identification of elevated uptake regions as physiological based on automated analysis of the sole CT information is particularly challenging for regions such as small intestines or ureters and would require manual corrections. A machine learning algorithm trained on multimodal PET/CT information may more accurately identify such regions of physiological uptake limiting the number of manual corrections required, as well as potentially being able to identify further challenging patterns of nonsuspicious uptake, such as uptake in ganglia and unspecific uptake in lymph nodes and bone. Recently, a convolutional neural network was trained with multimodal PET/CT information to identify tracer uptake regions suspicious for prostate cancer within the pelvis [25] with promising results.

In the current analysis, we developed and evaluated a multi-task convolutional neural network trained on the PET and CT information for the identification and anatomical location classification of suspicious tracer uptake sites in the entire axial body coverage of the scan. We employ multi-task training, previously evaluated in ^18^F-FDG PET/CT [26] with encouraging results, for assessment of ^68^Ga-PSMA-11 images. In addition, we explore two strategies to leverage training information from both radiotracers: transfer learning by pretraining on ^18^F-FDG images with fine-tuning on ^68^Ga-PSMA-11 images and a modified network architecture for synergistic dual-tracer learning. Moreover, we assess the ability of the trained network to support prostate cancer staging by evaluating its performance in automatically determining the N and M stage according to the PROMISE miTNM [14] framework.

## Materials and methods

### Patients

Two groups of subjects who underwent ^68^Ga-PSMA-11 PET/CT at the Klinikum rechts der Isar (Technical University of Munich) were retrospectively analyzed. The rationale for the definition and inclusion of the two groups was to allow the representation of different disease stages in the training dataset while keeping an acceptable expected annotation workload for the expert readers, employing a different annotation scheme for each group. The first group, referred to as group A, consisted of 123 consecutive subjects referred to PSMA-ligand PET/CT for primary staging or for assessment of biochemical recurrence. The second group, referred to as group B, consisted of 50 consecutive subjects referred to PSMA-ligand PET/CT for all indications of prostate cancer. PET/CT images were acquired on a Biograph mCT scanner (Siemens Medical Solutions). Diagnostic CT scans were acquired after intravenous injection of contrast agent (Imeron 300), followed by PET acquisition. PET scans were acquired 54 ± 10 min (mean ± std) after injection of ^68^Ga-PSMA-11 ligand solution (149 ± 26 MBq), with acquisition time of 3–4 min per bed position.

### Image analysis

#### Data annotation

PET/CT images were reviewed by expert nuclear medicine physicians who segmented sites of elevated tracer uptake, labeled them as nonsuspicious or suspicious for prostate cancer, and assigned them an anatomical localization from a set of physiological uptake sites and sites relevant for staging. Due to differences in patient tumor burden and to maintain an acceptable annotation workload, different annotation schemes were used for subjects in group A and group B, which were then considered in the deep learning model development and validation. For subjects in group A, having a low tumor burden, all regions of elevated tracer uptake were segmented semi-automatically using 45% of region SUVmax thresholding [19]. For subjects in group B, which included cases of high tumor burden, all high-uptake sites with SUVmax above the average liver uptake within a PERCIST-based reference region [27] were segmented with an incremental connected component algorithm [28] using 45% of SUVmax thresholding, of which up to one hundred sites per subject with the highest SUVmax were annotated. For each subject in group B, at least ten suspicious uptake sites were annotated when present, additionally labeling sites with lower SUVmax if necessary.

#### Model development

Subjects of group A (n = 123) were assigned to an N and M stage based on expert annotations and following the PROMISE miTNM framework. A stratified split of subjects in group A based on stage was then performed forming a development (n = 71) and a hold-out test set (n = 52). All subjects of group B (n = 50) were added to the development set. Four-fold cross validation on the final development set (n = 121) was used to evaluate different model training schemes. The hold-out test set was used exclusively to report results of the model testing and was not employed for the model development. A diagram summarizing the dataset split is reported in Supplemental Fig. 1.

**Fig. 1:**
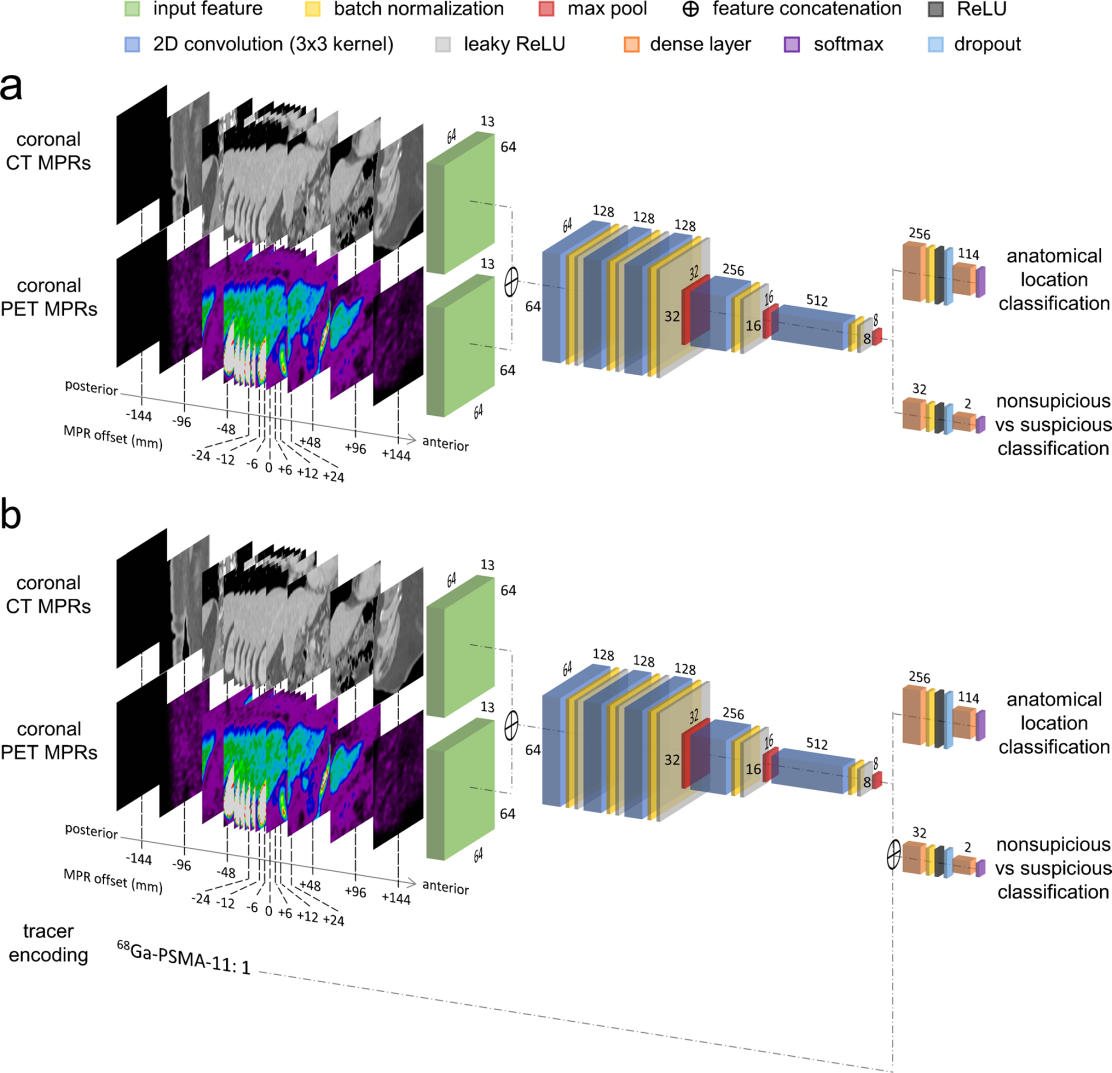
Convolutional neural network architecture used for PET uptake classification when training **a** with data from a single radiotracer and **b** with data from two radiotracers, encoding the tracer type as input to the network. Multiplanar reformations (MPRs) extracted from a region of interest being classified are represented as exemplar input to the network. In the three-dimensional illustration, numbers along layers’ edges indicate the size of the feature maps resulting as output of the corresponding layers

A multi-task convolutional neural network was trained to both classify PET/CT regions of interest as uptake suspicious or nonsuspicious for cancer and assign them an anatomical location classification. In addition to expert-annotated findings, regions of interest with SUVmax above 1 which were not labeled by the experts as suspicious were generated automatically with an incremental connected component algorithm [28], labeled as nonsuspicious, and used for training. These were generated using 45% of SUVmax thresholding and only for subjects in group A or subjects in group B with up to nine suspicious findings, i.e., for PET/CT images where all suspicious findings were annotated and remaining image regions could be considered physiological uptake. The network architecture and hyperparameters are illustrated in Fig. 1a. Inputs to the network are thirteen PET/CT coronal (192 mm × 192 mm) reformations extracted with offsets (− 144, − 96, − 48, − 24, − 12, − 6, 0, + 6, + 12, + 24, + 48, + 96, + 144 mm) from the region of interest SUVmax position, after resampling of PET and CT at 3 mm isotropic resolution, PET windowing between 0 and 15 SUV and CT windowing between − 300 and 300 HU.

We evaluated different training strategies to improve the algorithm performance. First (I), the model was trained with sequential sampling of all the training examples. Second (II), a balanced sampling of the training examples was performed, where a fixed maximum number of training examples per class per subject was randomly sampled at each training epoch (maximum of 32 physiological and 32 suspicious findings, 4 findings for each anatomical location class). Third (III), regions of interest used for training were augmented through affine transformations of the PET/CT randomly generated at each training epoch with isotropic scaling between 0.8 and 1.2 and rotations between − 17.2 and 17.2 degrees in all directions, to obtain additional regions with plausible pose and size. Forth (IV), to leverage expert knowledge of the same task in ^18^F-FDG PET/CT images, we trained the network as in (III) with datasets from [26], with a single split between training (90%) and validation (10%). The rationale for the ^18^F-FDG dataset split was to maximize the training data for knowledge transfer to PSMA-ligand PET/CT, while evaluation on a hold-out ^18^F-FDG test set was considered outside the scope of the analysis, which is mainly focused on assessing the proposed method for staging support in PSMA-ligand PET/CT. Fifth (V), we evaluated transfer learning by fine tuning on PSMA-ligand PET/CT data the network weights initially trained with ^18^F-FDG PET/CT images. Sixth (VI), we evaluated simultaneous training with PSMA-ligand and FDG PET/CT images by adding a binary input encoding the tracer type to the first fully connected layer and only for the output branch of the network responsible for classifying nonsuspicious vs. suspicious uptake, as illustrated in Fig. 1b.

#### Model testing

We validated the network with the highest performance by training it on the entire development set and evaluating it on the test set. In addition, we assessed the ability of the algorithm to determine the N and M stage from ^68^Ga-PSMA-11 PET/CT images fully automatically. For each test set subject, we first segmented all regions with SUVmax above 1 using an incremental connected component algorithm [28] and 45% of SUVmax thresholding. These regions were then processed by the convolutional neural network, classified as nonsuspicious or suspicious, and assigned an anatomical location label. The anatomical location labels of regions classified as suspicious were used to obtain a prediction of the N and M stage according to the PROMISE miTNM framework. Following PROMISE recommendations, a distinction between patterns of bone metastases was considered. No subject presented diffuse bone marrow involvement. This resulted in three N stage categories, related to regional lymph node metastases: N0 (none), N1 (single), N2 (multiple); and six M stage categories, related to distant metastases: M0 (none), M1a (extrapelvic lymph nodes), M1b/u (single bone lesion, unifocal), M1b/o (up to three multiple bone lesions, oligometastatic), M1b/d (four or more bone lesions, disseminated), M1c (other organs). Predictions of the N and M stage were then compared to the ones based on expert annotations.

### Statistical analysis

The main metrics used to evaluate the network performance were the area under the precision-recall curve, which accounts for marked class imbalance, referred to as average precision (AP), for the classification of regions as suspicious or nonsuspicious, and the classification accuracy of regions labeled as suspicious by the experts, for the anatomical location classification. The performance metrics were evaluated by pooling findings of all subjects together, and a 95% confidence interval was calculated by 2000 bootstrap resampling of the subjects. To compare different training schemes on the development set, a two-sided paired z-test was performed based on the bootstrap replicates with a significance level set to 5%. Bonferroni correction was used to account for multiple comparisons. For the test set, additional performance metrics were evaluated: number of true positives, false positives, false negatives, recall and positive predictive value for the classification as suspicious or nonsuspicious, classification accuracy of all findings labeled by the experts for the anatomical location classification. For the test set, except for the average precision, the performance metrics were also evaluated and reported on a per-subject basis. Agreement between the N and M stage estimated using the CNN and determined from the expert labels was assessed using percent agreement and confusion matrices.

## Results

In total, 173 subjects were included in the analysis of which 123 in group A and 50 in group B. A total of 5,577 high uptake regions were annotated, of which 4,520 were physiological uptake and 1,057 were suspicious uptake. The median volume of regions annotated as suspicious was 1.3 ml (interquartile range 0.6–3.0 ml). In addition to the expert-annotated findings, more than 160,000 regions with nonsuspicious uptake were automatically generated for subjects in the development set. A summary of the findings and expert annotations is reported in Supplemental Table 1. Based on the expert annotations of subjects in group A, 52 patients had miN0 stage, 40 had miN1 stage, and 31 had miN2 stage, whereas 41 subjects had miM0 stage, 21 had miM1a stage, 57 had miM1b stage, and 4 had miM1c stage. A summary of the N and M stage for subjects in group A is reported in Supplemental Table 2.

**Fig. 2:**
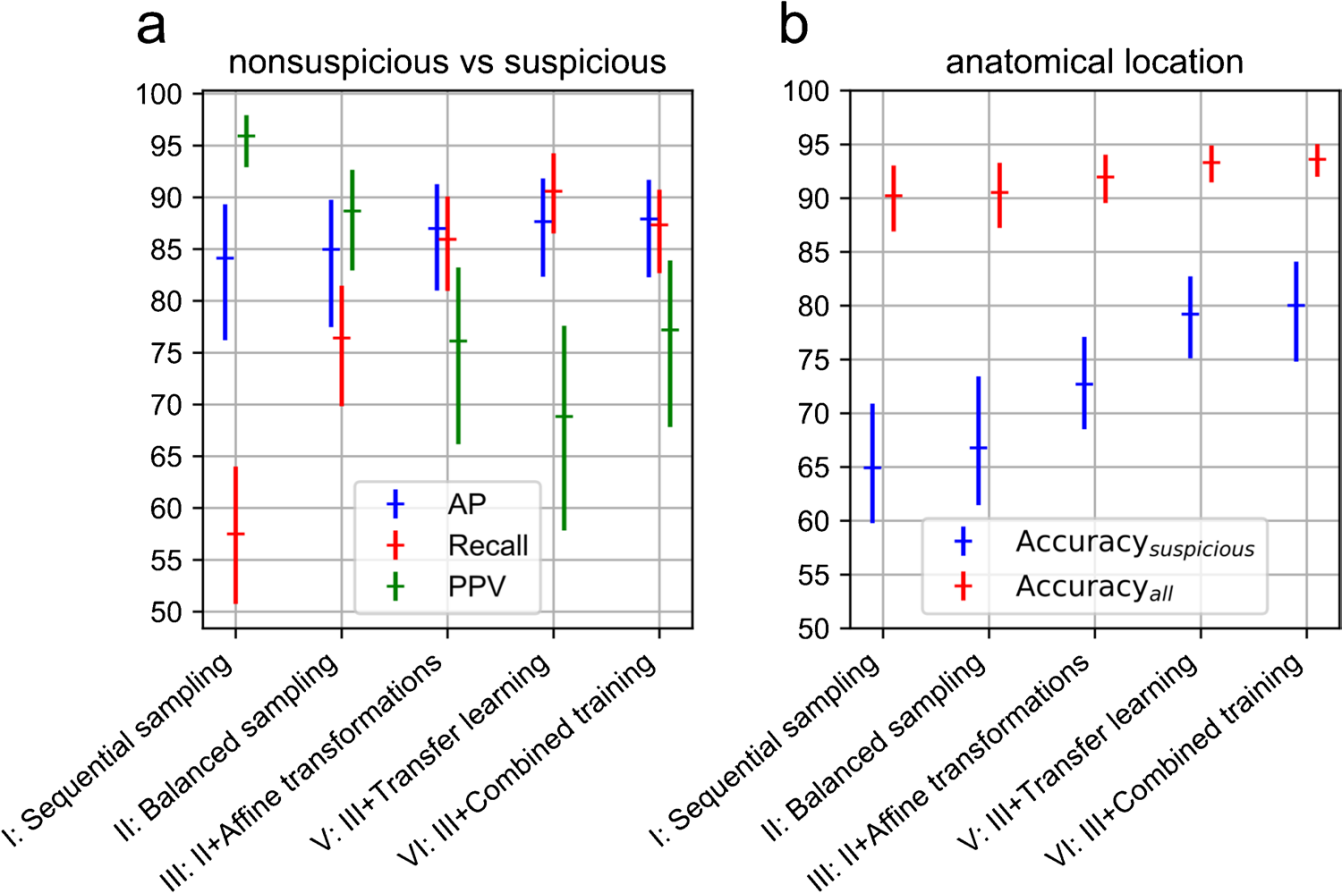
Performance obtained for **a** classification of PET uptake sites as nonsuspicious or suspicious and **b** classification of their anatomical location, using different strategies to train a convolutional network evaluated with four-fold cross validation on the development set of ^68^Ga-PSMA-11 PET/CT scans. Performance metrics are determined by pooling findings of all subjects together. Error bars indicate the 95% confidence interval obtained via bootstrap resampling at subject level

Figure 2 illustrates results obtained using different methods to train the CNN, evaluated with cross validation on the development dataset of ^68^Ga-PSMA-11 PET/CT images. The corresponding main performance metrics are summarized in Table 1. For the classification of findings as suspicious or nonsuspicious, using sequential sampling (I) as baseline [AP: 84.1, confidence interval (CI): 76.2–89.3], a performance improvement not statistically significant after applying Bonferroni correction was found with other training schemes including balanced sampling (II) (AP: 85.0, CI: 77.5–89.8, p = 0.197), its combination with affine (III) data augmentation (AP: 87.0, CI: 81.0–91.3, p = 0.067), and their combination with transfer learning (V) (AP: 87.7, CI: 82.3–91.8, p = 0.072) or combined training with ^18^F-FDG data (VI) (AP: 87.9, CI: 82.3–91.7, p = 0.047). Balanced sampling allowed markedly lower training time due to fewer training examples being processed on average per epoch (3584 vs. 128,640). For the anatomical location classification of suspicious findings, compared to sequential (I) sampling (accuracy: 64.9, CI: 59.8–70.9), affine data augmentation (III) significantly improved performance (accuracy: 72.7, CI: 68.5–77.1, p < 0.001) while balanced sampling (II) alone did not (accuracy: 66.8, CI: 61.5–73.4, p = 0.095). Compared to affine data augmentation (III), transfer learning (V) showed a further significant improvement (accuracy: 79.2, CI: 75.1–82.7, p = 0.001), with a performance not significantly different compared to combined training with ^18^F-FDG data (VI) (accuracy: 80.0, CI: 74.8–84.1, p = 0.489), which overall scored highest for both classification tasks.

**Table 1:** PET uptake classification performance using different strategies to train a convolutional network, evaluated with four-fold cross validation on a development set of ^68^Ga-PSMA-11 PET/CT scans and a fixed validation dataset of ^18^F-FDG PET/CT scans. Performance metrics are determined by pooling findings of all subjects together, with a 95% confidence interval obtained via bootstrap resampling at subject level, reported in brackets. The p value for a two-sided paired z-test based on bootstrap replicates is reported

Tracer	^68^Ga-PSMA-11		^18^F-FDG	
Classification output	Nonsuspicious vs. suspicious	Anatomical location	Nonsuspicious vs. suspicious	Anatomical location
Performance metric	AP^a^	Accuracy_suspicious_	AP^a^	Accuracy_suspicious_
Model training				
I: Sequential sampling	84.1 (76.2, 89.3)	64.9 (59.8, 70.9)	-	-
II: Balanced sampling	85.0 (77.5, 89.8) p = 0.197 vs. I	66.8 (61.5, 73.4) p = 0.095 vs. I	-	-
III: II + Affine transformations	87.0 (81.0, 91.3) p = 0.067 vs. I	72.7 (68.5, 77.1) p < 0.001* vs. I	-	-
IV: III on ^18^F-FDG data	-	-	77.1 (70.8, 87.3)	76.7 (71.2, 81.3)
V: Transfer learning by fine tuning of IV	87.7 (82.3, 91.8) p = 0.072 vs. I	79.2 (75.1, 82.7) p = 0.001* vs. III	-	-
VI: III + combined training on ^68^Ga-PSMA-11 and ^18^F-FDG data	87.9 (82.3, 91.7) p = 0.047 vs. I	80.0 (74.8, 84.1) p = 0.489 vs. V	77.9 (71.0, 86.5) p = 0.739 vs. III	77.2 (71.1, 81.2) p = 0.681 vs. III

^a^Average precision

*Significant

**Fig. 3:**
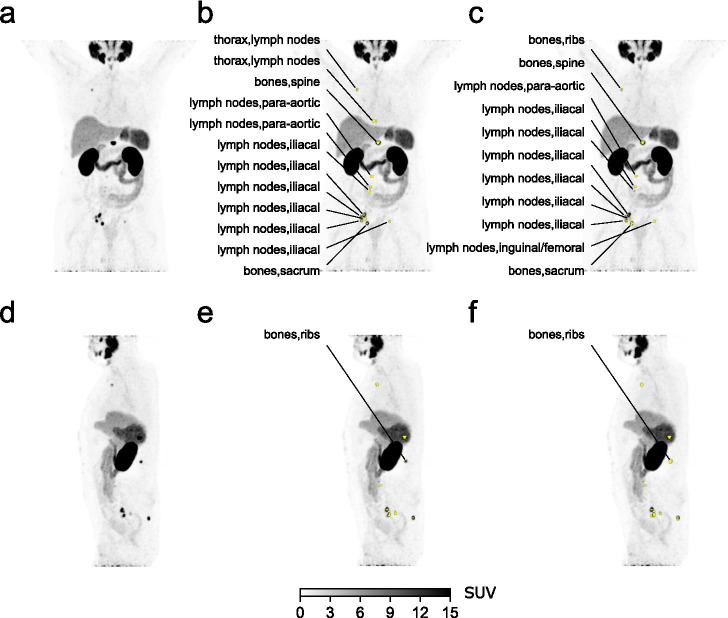
**a** Coronal and **d** sagittal maximum intensity projections (MIP) of a ^68^Ga-PSMA-11 PET scan for a subject in the test set. **b**, **e** Regions of interest classified by the convolutional neural network as suspicious overlayed to the PET MIP in yellow, together with the anatomical location label assigned by the network. **c**, **f** Regions of interest identified by an expert physician as suspicious uptake overlayed to the PET MIP in yellow, together with the anatomical location label assigned by the expert

Following combined training using ^18^F-FDG images together with ^68^Ga-PSMA-11 scans of the entire development set and evaluation on the ^68^Ga-PSMA-11 test set, an average precision of 80.4 (CI: 71.1–87.8), a sensitivity of 81.1% (CI: 70.6–90.1), and a positive predictive value of 66.8% (CI: 60.3–72.7) were obtained (Table 2). Anatomical location classification accuracy was 77.0% (CI: 70.0–83.4) for suspicious regions and 94.4% (92.4–96.1) for all expert-annotated regions. Figure 3 shows an example subject in the test dataset assessed using the CNN. After assigning an N stage based on CNN annotations and based on expert annotations, agreement was 67%, while agreement for identification of any pelvic nodal involvement (N0 vs. N1/N2) was 81%. The confusion matrix for the N stage assessment is shown in Table 3. After assigning an M stage based on CNN annotations and based on expert annotations, agreement was 62%, agreement excluding discrimination of bone involvement pattern was 73%, and agreement for identification of any distant metastases (M0 vs. M1) was 77%. The confusion matrix for the M stage assessment is shown in Table 4.

**Table 2:** PET uptake classification performance obtained with combined training of a convolutional neural network using ^68^Ga-PSMA-11 PET/CT and ^18^F-FDG PET/CT scans, evaluated on a hold-out test dataset of ^68^Ga-PSMA-11 PET/CT scans. Performance metrics determined by pooling findings of all subjects together are reported. Summary statistics for performance metrics determined at per-subject level are also reported. 95% confidence intervals obtained via bootstrap resampling at subject level are reported in brackets

Tracer	^68^Ga-PSMA-11						
Classification output	Nonsuspicious vs. suspicious						
Summary statistic	Pooled (CI)	Per-subject					
		Average	Min	Q1	Median	Q3	Max
Performance metric							
AP^a^	80.4 (71.1, 87.8)	-	-	-	-	-	-
Recall	81.1 (70.6, 90.1)	85.2	11.1	73.8	100.0	100.0	100.0
PPV^b^	66.8 (60.3, 72.7)	65.5	0.0	50.0	68.3	100.0	100.0
True positives	159 (114, 209)	3.1	0	1	2	3	19
False positives	79 (58, 102)	1.5	0	0	1	2	8
False negatives	37 (18, 59)	0.7	0	0	0	1	8
Classification output	Anatomical location						
Performance metric							
Accuracy_suspicious_	77.0 (70.0, 83.4)	78.4	0.0	57.5	95.0	100.0	100.0
Accuracy_all_	94.4 (92.4, 96.1)	94.1	78.6	90.9	94.9	100.0	100.0

^a^Average precision

^b^Positive predictive value

**Table 3:** Confusion matrix comparing the N stage determined according to the PROMISE miTNM framework based on expert annotations and based on convolutional neural network annotations

	Predicted stage			
	miN0	miN1	miN2	Total
Annotations stage				
miN0	14	7	1	22
miN1	2	11	4	17
miN2	0	3	10	13
Total	16	21	15	52

**Table 4:** Confusion matrix comparing the M stage determined according to the PROMISE miTNM framework based on expert annotations and based on convolutional neural network annotations

	Predicted stage						
	miM0	miM1a	miM1b/u	miM1b/o	miM1b/d	miM1c	Total
Annotations stage							
miM0	8	5	4	0	0	0	17
miM1a	2	6	1	0	0	0	9
miM1b/u	1	0	7	4	0	1	13
miM1b/o	0	0	0	3	1	0	4
miM1b/d	0	0	0	1	6	0	7
miM1c	0	0	0	0	0	2	2
Total	11	11	12	8	7	3	52

## Discussion

In this analysis, we showed that a convolutional neural network can be trained to classify sites of elevated ^68^Ga-PSMA-11 uptake in the entire axial body coverage of the scan by leveraging both PET and CT information. Having extensively included in the training data regions with uptake above 1 SUV, the network can be used to assess a broad window of the tracer distribution in the body and effectively identify sites suspicious for prostate cancer. Moreover, thanks to the combined identification of suspicious uptake sites and the classification of their anatomical location, the network can be used to assess the spread pattern of suspicious sites in different organs and tissues and was able to determine the N and M stage according to the PROMISE miTNM framework in good agreement with the expert evaluation. Additionally, we found that including training information from PET/CT images and expert annotations obtained with a different PET tracer improved the network performance on ^68^Ga-PSMA-11 PET/CT images, for which a limited number of reader-annotated cases were available. Previously described methods for ^68^Ga-PSMA-11 PET/CT image analysis using machine learning were trained on PET/CT information to identify suspicious uptake regions limited to the pelvis [25] or were trained on CT-only information to segment a predefined set of organs and then used to guide semi-automated identification of suspicious high uptake regions in the whole body [18, 24].

In the current analysis, regions of interest were segmented both by the expert reader as well as for the network training and validation using methods based on thresholding, which allow limited flexibility and accuracy in delineating contours. Although the threshold-based segmentation methods used have limited accuracy, these offer a practical solution for rapid semi-automated annotation by an expert reader, they are often used in clinical practice and research studies on metabolic tumor volume [29], as well as mentioned in procedure guidelines [30]. Nonetheless, efforts for standardizing and advancing segmentation techniques are ongoing, and machine learning-based methods are promising for improving automated segmentation accuracy. Notably, for tumor segmentation in ^18^F-FDG PET/CT images, machine learning methods have recently shown improved test–retest repeatability [31] and accuracy [32, 33] compared to thresholding methods, as well as ability to delineate tumor regions in the whole body [34, 35]. While our results with ^68^Ga-PSMA-11 PET/CT images support the use of machine learning methods for identification and anatomical location classification of suspicious uptake sites, future analyses are required to evaluate the accuracy and repeatability of different segmentation methods in PSMA-ligand images for varying tumor sites in the whole body. Moreover, while different software implementations of threshold-based segmentation methods were reported to yield comparable results for metabolic tumor volume quantification in ^68^Ga-PSMA-11 PET/CT scans [36], there may be variations in machine learning-based segmentation methods and the concordance and potential standardization of these should also be investigated.

A limited number of subjects with advanced prostate cancer were included in the analysis and these were used solely for the network training. Given the very low tumor burden of subjects in the test set, it was not possible within the context of this analysis to evaluate the ability of the proposed method to estimate total tumor volume within a wide range and in particular for subjects at an advanced stage, for which tumor burden may be more informative. Furthermore, the majority of uptake regions annotated as suspicious for prostate cancer were in lymph nodes or bone, while suspicious findings in other organs were limited. Since the network was trained to evaluate single regions of interest, it was possible to use PET/CT scans with only partial annotation of suspicious sites for training. This is beneficial since labeling can be highly time consuming in cases where a large number of lesions need to be fully annotated. Moreover, as the network was trained with a variety of regions of interest in the whole body, it may prove useful for the staging and tumor burden assessment also in subjects with an advanced disease, but this will need to be confirmed in future analyses.

The ground truth used to train and evaluate the proposed algorithm was determined by visual assessment of the images by an expert physician, while neither histopathology nor follow-up information was considered. Additionally, PET/CT image quality characteristics, such as pitfalls due to motion or artifacts, reconstruction settings, and partial volume effects may influence the output of the network and results will require expert supervision for the use in clinical context. Despite the above limitations, the network showed good ability to identify even small suspicious sites with a limited number of false positives, compared to the expert evaluation.

In this analysis, despite the fact that the ^18^F-FDG PET/CT datasets were included from a previous investigation and not specifically selected for the present survey, we found that combining training information from ^18^F-FDG PET/CT and ^68^Ga-PSMA-11 PET/CT led to improved accuracy for the identification and anatomical location classification of suspicious uptake sites. This result brings forward the promising perspective of a deep learning framework for supporting staging and tumor burden assessment in multiple cancer types with PET/CT images obtained using different tracers. Notably, an increasing variety of PET radiotracers is being clinically used and developed in oncology, with multiple alternative compounds undergoing clinical trials for PSMA-targeted imaging alone. On the one hand, the lesion anatomical spread pattern and tumor volume are meaningful biomarkers in different cancer types independently of the PET tracer used. On the other hand, with each compound having a different biodistribution, training distinct networks de novo as a separate solution for each tracer would require a significant number of image datasets and expert annotations to reach sufficient accuracy. Ideally, combining information from multiple diseases and tracers in a single network could create synergies, leveraging similarities in physiological uptake, tracer excretion patterns, and tumor spread, while still accounting for differences based on the provided input encoding the tracer type. In the current analysis, performance improvements when training with information from both ^68^Ga-PSMA-11 PET/CT and ^18^F-FDG PET/CT images were found mainly for ^68^Ga-PSMA-11 PET/CT scans, having a smaller training dataset. Moreover, a significant improvement was found for the task of anatomical location classification, possibly driven mainly by the larger CT training information, while the performance increase in identification of suspicious uptake was less pronounced. The overall benefit of a combined training approach may depend on the level of similarity and the relative frequency of the different imaging findings between tracers, and future analyses will be required to evaluate the extensibility of the proposed framework to additional patient cohorts and radiotracers.

## Conclusion

The evaluated convolutional neural network showed good agreement with expert assessment for identifying sites of suspicious uptake in whole-body ^68^Ga-PSMA-11 PET/CT, assigning them an anatomical location classification, and determining the N and M stage according to a standardized framework. Both transfer learning and combined training using ^18^F-FDG PET/CT images and expert annotations improved performance. The investigated techniques are promising for enabling efficient assessment of tumor spread and overall burden with established and novel tracers, considering the limited availability of expert-annotated ground truth.

## Supplementary Information

Below is the link to the electronic supplementary material.

Supplementary file1 (DOCX 245 KB)

## Data Availability

The software implementing the convolutional neural network can be made available for distribution through a collaboration agreement by contacting the corresponding author.
