# Supplementary material for: Whole-body uptake classification and prostate cancer staging in 68Ga-PSMA-11 PET/CT using dual-tracer learning
Source: Eur J Nucl Med Mol Imaging. 2021 Jul 7;49(2):517–26. doi: 10.1007/s00259-021-05473-2 (PMC8803695; doi:10.1007/s00259-021-05473-2)
Supplement: Supplementary file 1 — Supplementary file1 (DOCX 245 KB) [file 259_2021_5473_MOESM1_ESM.docx]

**SUPPLEMENTARY INFORMATION**

**Supplemental Fig. 1** Diagram summarizing the PET/CT datasets used in the analysis and the data split performed for the deep learning model development and testing


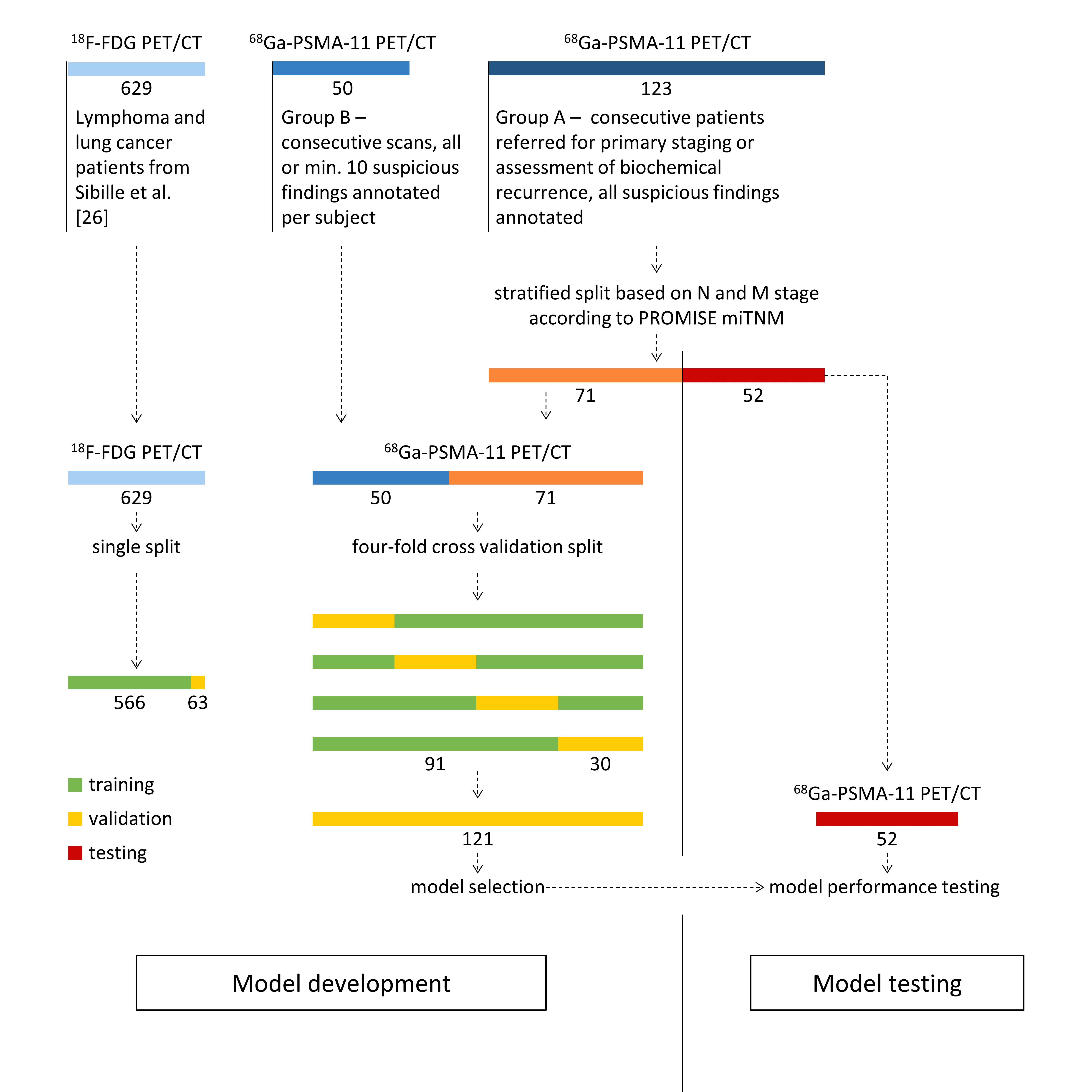


Supplemental Table 1: Summary of the findings annotated by an expert reader in ^68^Ga-PSMA-11 PET/CT images using semi-automated segmentation methods, reported by anatomical location label assigned, in descending order of occurrence.

|  | **Number of findings** | **Number of suspicious findings (%)** |
| --- | --- | --- |
| Total | 5577 | 1057 (19) |
| **Anatomical location** |  |  |
| abdomen, liver | 1875 | 22 (1) |
| abdomen, small intestine | 870 | 8 (1) |
| abdomen, kidney | 357 | 0 (0) |
| neck, submandibular gland | 334 | 0 (0) |
| neck, parotid gland | 310 | 0 (0) |
| abdomen, spleen | 277 | 0 (0) |
| abdomen, lymph nodes, iliacal | 164 | 159 (97) |
| neck, sublingual gland | 149 | 0 (0) |
| abdomen, bladder | 146 | 0 (0) |
| abdomen, lymph nodes, para-aortic | 134 | 133 (99) |
| abdomen, bones, pelvis | 123 | 121 (98) |
| thorax, bones, spine | 109 | 109 (100) |
| abdomen, bones, spine | 86 | 77 (90) |
| thorax, lymph nodes | 79 | 75 (95) |
| thorax, bones, ribs | 72 | 72 (100) |
| neck, glottis | 50 | 0 (0) |
| abdomen, bones, sacrum | 50 | 47 (94) |
| abdomen, ureter | 38 | 1 (3) |
| abdomen, prostate | 30 | 30 (100) |
| abdomen, lymph nodes, presacral | 29 | 28 (97) |
| neck, tonsils | 27 | 0 (0) |
| lower limb, bones, femur | 23 | 22 (96) |
| thorax, bones, scapula | 23 | 23 (100) |
| neck, bones, spine | 20 | 19 (95) |
| cranium, nose | 17 | 0 (0) |
| thorax, bones, sternum | 17 | 17 (100) |
| abdomen, lymph nodes, obturator | 15 | 15 (100) |
| neck, thyroid | 15 | 0 (0) |
| neck, cervical lymph nodes | 13 | 11 (85) |
| upper limb, bones, humerus | 12 | 12 (100) |
| abdomen, lymph nodes, inguinal | 10 | 9 (90) |
| thorax, lung | 10 | 9 (90) |
| cranium, mouth, teeth | 9 | 0 (0) |
| abdomen, colon | 8 | 0 (0) |
| abdomen, lymph nodes, inguinal / femoral | 8 | 8 (100) |
| thorax, oesophagus | 7 | 0 (0) |
| thorax, bones, clavicle | 6 | 6 (100) |
| thorax, skin | 5 | 5 (100) |
| abdomen, ganglia | 5 | 1 (20) |
| abdomen, rectum | 4 | 2 (50) |
| cranium, skull | 4 | 3 (75) |
| thorax, ganglia | 4 | 1 (25) |
| cranium, mouth, palate | 4 | 0 (0) |
| lower limb, lymph nodes, femoral | 3 | 3 (100) |
| cranium, mouth, floor of mouth | 3 | 0 (0) |
| abdomen, skin | 3 | 2 (67) |
| cranium, eye | 3 | 0 (0) |
| abdomen, lymph nodes, mesenterial | 3 | 2 (67) |
| abdomen, lymph nodes, peri-hepatic | 2 | 1 (50) |
| thorax, mediastinum, hilum | 2 | 0 (0) |
| abdomen, penis | 2 | 0 (0) |
| abdomen, testis | 2 | 0 (0) |
| abdomen, adrenal gland | 1 | 1 (100) |
| neck, accessory sinuses, maxillary sinus | 1 | 0 (0) |
| thorax, pleura | 1 | 1 (100) |
| abdomen, stomach, cardia / fundus / body | 1 | 0 (0) |
| abdomen, lymph nodes, peri-splenic | 1 | 1 (100) |
| neck, bones, clavicle | 1 | 1 (100) |

Supplemental Table 2 Summary of the N and M stage assigned based on expert reader annotation of PSMA-ligand PET/CT images according to the PROMISE miTNM framework, for subjects in group A.

|  | **Development (group A)** | **Test** |
| --- | --- | --- |
| Total | 71 | 52 |
| **Stage** |  |  |
| miN0M0 | 8 | 6 |
| miN1M0 | 11 | 8 |
| miN2M0 | 5 | 3 |
| miN0M1a | 2 | 1 |
| miN1M1a | 3 | 3 |
| miN2M1a | 7 | 5 |
| miN0M1b/u | 11 | 8 |
| miN1M1b/u | 7 | 4 |
| miN2M1b/u | 1 | 1 |
| miN0M1b/o | 3 | 2 |
| miN1M1b/o | 1 | 1 |
| miN2M1b/o | 2 | 1 |
| miN0M1b/d | 5 | 4 |
| miN1M1b/d | 1 | 1 |
| miN2M1b/d | 2 | 2 |
| miN0M1c | 1 | 1 |
| miN2M1c | 1 | 1 |
